# Significant Shrinkage of Multifocal Liver Metastases and Long-Term Survival in a Patient With Rectal Cancer, After Trans-Arterial Chemoembolization (TACE): A Case Report

**DOI:** 10.1097/MD.0000000000001848

**Published:** 2015-10-23

**Authors:** Bogdan Andrei Suciu, Simona Gurzu, Lucian Marginean, Doina Milutin, Ioana Halmaciu, Ioan Jung, Klara Branzaniuc, Calin Molnar

**Affiliations:** From the Department of Surgery, University of Medicine and Pharmacy of Tirgu-Mures, Tirgu-Mures, Romania (BAS, CM); Department of Anatomy and Embryology, University of Medicine and Pharmacy of Tirgu-Mures, Tirgu-Mures, Romania (BAS, IH, KB); Department of Pathology, University of Medicine and Pharmacy of Tirgu-Mures, Tirgu-Mures, Romania (SG, DM, IJ); and Department of Radiology, University of Medicine and Pharmacy of Tirgu-Mures, Tirgu-Mures, Romania (LM, IH).

## Abstract

In this paper, we present the successful therapeutic approach of unresectable liver metastases in a patient with rectal cancer.

A 63-year-old male underwent endoscopic polypectomy followed by rectosigmoid resection for an adenocarcinoma of the rectum diagnosed in pT2N0 stage. The angio-computed tomography (CT) revealed four metastatic hepatic nodules ranging from 12 to 130 mm in diameter. After one cure of trans-arterial chemoembolization (TACE) with lipiodol and 5-fluorouracil, combined with FOLFOX4 + capecitabine systemic chemotherapy, the diameter of all hepatic nodules decreased to half size, at 6 months after TACE. Further curative surgical hepatic metastasectomy was done and complete pathologic response was obtained. The patient is free of recurrences and metastases after 26 months of follow-up.

This representative case shows that an efficient trans-disciplinary approach could lead to successful therapeutic management even in patients with advanced-staged colorectal carcinomas.

## INTRODUCTION

Colorectal cancer (CRC) is the third commonest cancer worldwide and about 15% to 30% of patients show liver metastases at the time of diagnosis.^1^ In previous reports, it was shown that curative liver resection can be practiced in only 10% to 15% of the patients with synchronous or metachronous CRC-related liver metastases.^2^

In patients with unresectable hepatic metastases from CRC (more than 4 metastases or metastatic nodules larger than 50 mm, bilobar character, invasion of pedicle lymph nodes, serum level of carcinoembryonic antigen (CEA) higher than 200 ng/mL), oxaliplatin-based systemic chemotherapy is mostly used but metastatic shrinkage is obtained in fewer than 10% to 15% of the patients.^3,4^ Trans-arterial chemoembolization (TACE) currently represents the standard loco-regional therapy, but its real efficacy is not yet known.^2,5^ It consists of an angiographic insertion, in the selected branches of the hepatic artery, of a catheter loaded with an embolizing emulsion consisting of lipiodol and a chemotherapic agent (oxaliplatin, irinotecan, 5-fluorouracil, etc.).^2,5,6^ Drug eluting bead chemoembolization (DEB-TACE) is a variant of the conventional TACE procedure in which lipiodol is replaced by polymer-based microparticles that are considered having a better ability for tumor penetration and a lower systemic toxicity,^6^ but few data are reported in this field.

In this report, we present a complete liver resectability, 6 months after TACE, in a patient with initial diagnosis of rectal carcinoma with unresectable liver metastases. Signed informed consent was preoperatively obtained to perform surgery and publish the case details. This is a case report and institutional review board approval was not necessary.

### Case Presentation

**FIGURE 1:**
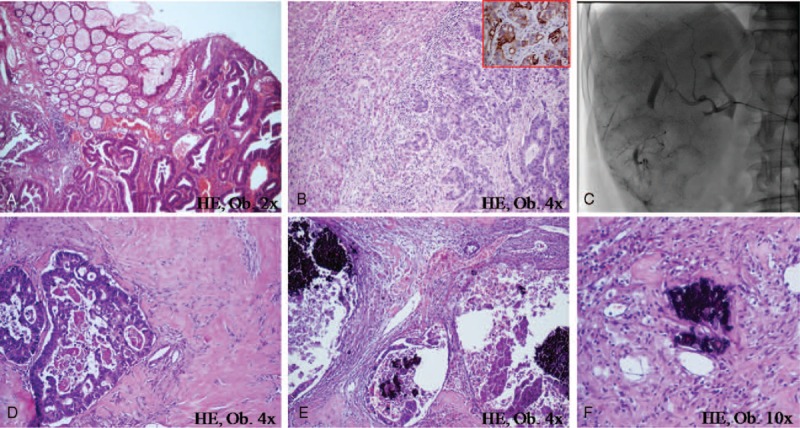
In a patient with rectal adenocarcinoma (A) and liver metastases (B) marked by keratin 20 (B-frame), at 6 months after TACE (C), large hyalinized areas (D), and necrosis (E) can be seen in the hepatic metastasectomy specimen. The chemotherapic blue crystals can be observed inside the glandular lumen (E) and intravascular (F).

**FIGURE 2:**
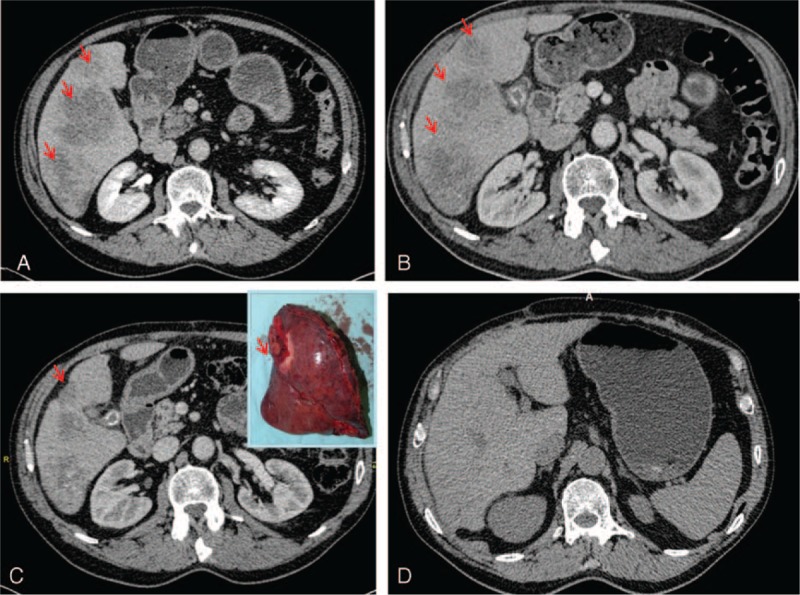
Compared to the preoperative aspect of the liver (A), the angio-CT shows shrinkage of the hepatic metastatic nodules at 3 months (B) and capsular contracture at 6 months after TACE (C), also noted on the surgical specimen (C-frame). No metastases are noted at 26 months after surgery (D).

A 63-year-old male was hospitalized with long-time history of fecal occult bleeding. The flexible sigmoidoscopy showed a polypoid lesion in the proximal rectum that was endoscopically resected. The tumor was diagnosed as a moderately differentiated adenocarcinoma developed on the background of an adenomatous polyp, with tumor infiltration of the pedicle (Fig. 1), and no possibility of evaluation of the basis of implantation. The abdominal angio-computed tomography (CT) examination showed four large hepatic metastases, from 12 to 130 mm (Fig. 2 and Table 1).

**TABLE 1:**
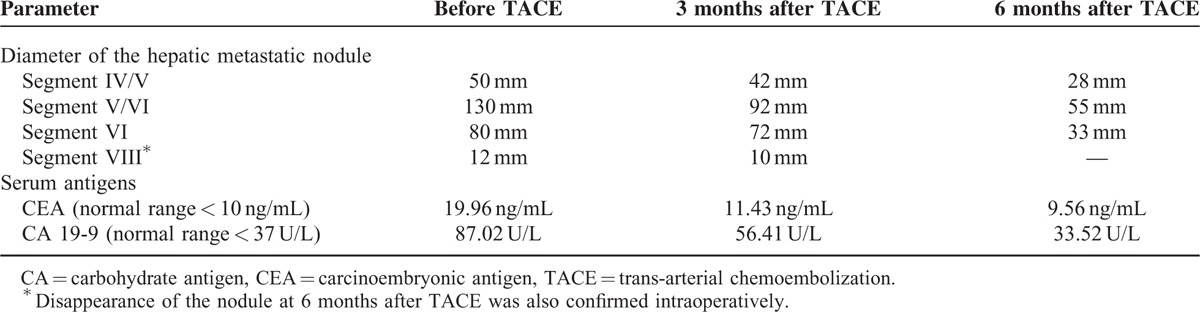
Angio-CT Evaluated Shrinkage of the Hepatic Metastatic Nodules and Progressively Normalization of the Serum Parameters in a Patient With Rectal Cancer Treated With TACE Combined With Systemic Chemotherapy

Rectosigmoid resection was done and the intraoperative exploration confirmed the multifocal unresectable hepatic metastases. Metastatic biopsy form the VIth segment of the liver was performed. Histological examination of the rectosigmoid showed no remnant tumor cells in the surgical specimen and absence of lymph node metastases. Based on the endoscopic specimen aspect, and keratin 20 positivity within the glandular structures from the liver nodule (Fig. 1), the tumor was finally diagnosed as “moderately differentiated adenocarcinoma of the rectum, pT2N0M1 stage.”

One month after surgery, the oncologic protocol started with 1 session of TACE, which was trans-femorally performed and consisted of infusion of 10 mL of lipiodol and 5-fluorouracil (1000 mg/24 hours) in the right branch of the hepatic artery (Fig. 1). No side effects were noticed except for a transient increase of serum level of transaminases. One month after TACE, 6 cycles of 14 days of systemic chemotherapy with oxaliplatin combined with 5-fluorouracil (FOLFOX4 protocol) and capecitabine (2500 mg/m^2^/day) were performed, according to the international guidelines.^7^

The control angio-CT performed at 3 and 6 months after TACE (Fig. 2) showed progressive shrinkage of the hepatic metastases volume, from 1.8 to 2.4 times, compared to the initial nodules and disappearance of the smallest metastatic nodule, as shown in Table 1. Because the patient clinical status was not modified, hepatic metastasectomy was decided. Atypical surgical resection of the hepatic segments IVB, V, and VI was performed (Fig. 2).

Gross findings of the resected hepatic specimen confirmed the peripheral fibrotic retraction in the area corresponding to the IVth segment. Under the microscope, malignant glandular structures with large necrotic areas surrounded by small round chemotherapic blue crystals, intra- and perivascular located, were noted (Fig. 1). The hepatic resection margins were negative.

The 6 cycles of systemic chemotherapy (FOLFOX4 + capecitabine), using the same therapeutic protocol, were repeated. The patient is alive at 26 months after first surgical intervention, with no signs of tumor recurrence or other metastases at control-CT (Fig. 2) and normal serum levels of CEA and carbohydrate antigen (CA) 19-9, as shown in Table 1.

## DISCUSSION

Despite progresses in oncology therapy, the 5-year survival rate of patients with unresectable liver metastases does not exceed 10% of the cases. However, in cases where shrinkage of the metastatic nodules can be obtained, with subsequent curative liver resection, the overall survival rate was reported to increase up to 25% to 40%.^2,8^

The most recent clinical trials using TACE combined with systemic therapy, as a therapeutic option for patients with unresectable hepatic metastases from CRC, reported encouraging results, compared with systemic therapy only, but data in the field are controversial.^2^ Compared to systemic therapy, not only was TACE seen to assure a high intratumor concentration of chemotherapy agent and subsequently intratumor hypoxia and necrosis, but also the chemotherapy-related systemic effects were reduced.^2^ Moreover, the rate of curative resectability significantly increases after TACE, compared with systemic therapy only, independent of the chemotherapic drugs used.^9^ Lipiodol is combined with the chemotherapic agent for its well-proved embolic effect but it was also proved that it can prolong the intratumor passage of the drug.^10^

The pathomechanism of TACE-related shrinkage of the tumor volume is mainly related to intratumor hypoxia and subsequently tumor necrosis with further hyalinization and contraction of the fibrotic tissue.^11^

Currently in the literature, there is only 1 randomized trial that evaluated the usefulness of TACE compared to systemic chemotherapy in patients with liver metastases after CRC, which shows no significant increased survival in the patients.^12^ However, this study took into accounts only 22 randomly selected patients, all of them presenting huge metastases (over 75% of the liver parenchyma was infiltrated).^12^ In other trials, significant TACE-related shrinkage of the metastatic nodules was reported in 42% to 62% of the patients, compared with 9% to 21% of the cases in which only systemic chemotherapy was used.^13,14^

The most recent report in the field was published by Pernot et al,^5^ the authors presenting the first case of complete pathologic response (CPR) obtained in a patient with a pT3N0 stage sigmoid carcinoma, with 6 unresectable hepatic metastases, similar to our case. In Pernot et al's patient, concomitant systemic FOLFOX4 combined with two sessions of drug-eluting beads loaded with irinotecan (DEBIRI) was performed,^5^ whereas 5-fluorouracil and systemic FOLFOX4 + capecitabine was the therapeutic regimen used in our case. After 2 sessions of embolization, they obtained CPR and proved under the microscope that no tumor cells were present in the hepatic parenchyma.^5^ In our case, significant shrinkage (from 130 to 55 mm) was obtained at 6 months after 1 session of embolization but the tumor cells did not disappear.

It is worth noticing that the CPR was microscopically appreciated by Pernot et al based on the presence of fibrosis and acellular mucoid lakes in the liver specimens, without mentioning the immunoprofile of these lakes (CEA and keratins). Moreover, they did not mention data about the CEA and CA 19-9 serum levels in the patient, before and after TACE.^5^

The same histological criteria were proposed by the College of American Pathologists to evaluate the CPR to preoperative chemoradiation in resected specimens of rectal cancer patients.^15^ According to these criteria, the presence of fibrotic areas with acellular mucin pools, in the specimens examined postradiochemotherapy, should be considered as a CPR, independent of the histological aspect of the primary tumor (mucinous carcinomas vs. adenocarcinomas without mucinous component).^15^ However, this aspect is controversial because the acellular pools can show CEA positivity correlated with a high serum CEA level.^16^ Moreover, in some rectal specimens with postchemoradiotherapy mucin lakes, tumor cells can be seen after multisliced section. Based on this fact, it was previously suggested by our team that “the presence of acellular mucin pools in surgical specimens of rectal cancers cannot be interpreted as an indicator of complete response at radiotherapy if at least 10 multilevel sections are performed in at least 3 tumor blocks per case, and CEA negativity is not proved.”^16^

This case is the first one reporting CPR based on the metastases’ complete resectability, without recurrences, and constant low serum levels of CEA and CA 19-9, at 24 months after TACE. The case confirms the promising TACE-related results, combined with neoadjuvant systemic chemotherapy, in patients with unresectable liver metastases after CRC, especially in cases without lymph node metastases.

*Limitations of the paper*: This paper was based on only 1 case with a follow-up time of 26 months. To prove the efficacy of TACE combined with neoadjuvant systemic chemotherapy, in patients with multifocal unresectable liver metastases after CRC, a longer follow-up in several cases would be necessary.
